# Making their minds up: flux and stability in young children’s career aspirations in North East England

**DOI:** 10.1057/s41599-025-05364-z

**Published:** 2025-07-08

**Authors:** Carol Davenport, Annie Padwick

**Affiliations:** Northumbria University, Newcastle upon Tyne, UK

**Keywords:** Education, Science, technology and society

## Abstract

School career information advice and guidance in England has typically focused on pupils aged 13–18. However, pupils aged under 11 have already formed career aspirations. Career aspirations are used as a proxy for future occupational destinations. This study tracks the individual career aspirations over 3 years for 78 children aged 7–9 at the start of study, from three schools in the North East England taking part in a STEM outreach project. The data are also used to explore the applicability of aspiration development frameworks for younger children. The majority of children were able to name at least one career aspiration with nearly 70% naming more, although these aspirations were drawn from a relatively narrow pool of jobs related to children’s interests and jobs they see around them. 38% of the children had the same aspiration over time, but 60% of children changed their careers aspirations completely over the 3 years of the study. Career aspirations were strongly gendered, with boys naming a smaller pool of jobs most often. Between 2019 and 2021, there was an increase in STEM aspirations named by boys, and a decrease by girls. Thematic analysis of the reasons given for different aspirations identified four themes: self-actualisation, altruism, characteristics of the job, and role models. These themes were related to the aspiration frameworks. This study shows that there is both flux and stability in children’s aspirations between the ages of 7 and 11. The gendered career choices at both time points indicate that there is a need for educators to challenge stereotypes about who can do what type of job from a much earlier age. All aspirations were drawn from a relatively small pool of job suggesting that introducing children to a wider range of jobs at an earlier age could support development of aspirations.

## Introduction

Statutory career information advice and guidance (CIAG) in schools in England has traditionally been focused on the decision-making points in a young person’s life, typically around the ages of 13–18 (Mulvey, 2006). In the last decade in England this age range has slowly been extended to reach younger children, first to 12-years-olds (DfE, 2014) and then to 11-year-olds (DfE, 2023). There is increasing support for the inclusion of CIAG type activities within primary schools in England (Millard et al., 2019; Careers & Enterprise Company, 2023) and evidence that such activities are taking place in some schools (Hughes et al., 2022; Davenport & Padwick, 2023; Careers & Enterprise Company, 2024).

There is a recognition in the literature that children start to make decisions about possible careers well before the age of 11 (Magnuson and Starr, 2000; Atherton et al., 2009). Aspirations are shaped by internalised social and cultural values, including gender role beliefs, i.e. the types of roles, responsibilities and behaviours deemed appropriate for men and women (Dicke et al., 2019), with the result that children’s interests and career aspirations are often delineated by gender from a young age (Gottfredson, 1981; Bian et al., 2017; Emembolu et al., 2020a). Traditional gender role beliefs about STEM persist (Dicke et al., 2019), where research indicates that children see maths and science, and particularly physics, as ‘for boys’ (Makarova et al., 2019), which has implications for their career aspirations (e.g. Archer et al., 2013). Where girls do have aspirations for STEM careers, they tend towards those related to health care and biology, whilst boys tend towards those related to physical sciences, engineering or skilled trades (Moote et al., 2020). Employer-focussed organisations have expressed concern about the gendered nature of children and young people’s career aspirations, particularly for careers in engineering and technology (Engineering UK, 2017; Chambers et al., 2018; Armitage et al., 2020), as well as the ‘unreality’ of young people’s aspirations when compared to the labour market (Mann et al, 2013, Rogers et al. 2020). This has led to calls to support children and young people to broaden their knowledge of different sectors and careers (Percy and Amegah, 2021).

The current career aspirations of children and young people have been studied as indicators of future occupational destinations, and although these aspirations may not precisely predict future aspirations, they are generally considered to be a useful proxy for career understanding (Rojewski, 2007). Atherton et al. (2009) asked pupils aged 11–12 about their career aspirations and 65% of the pupils said they had known the job they wanted to do in the future for at least 2 years, indicating some stability in their aspirations over that time period. Other studies also suggest that there is a level of stability in the aspirations of young people towards particular fields within STEM (e.g. Berger et al., 2019; Moote et al, 2020; Archer et al., 2020). Some STEM engagement interventions use changes in aspirations, or willingness to consider particular careers, as a way to measure the impact of the intervention (see e.g. Parker et al., 2020; Edmonds et al., 2022). However, without greater understanding of how the career aspirations of individuals might change over time, as opposed to changes at a cohort level, it is challenging to interpret measured changes or link them to the impact of an intervention.

The current paper presents findings from a longitudinal study of young children’s career aspirations gathered as part of a 3-year STEM outreach project. Given the expansive range of influences on aspirations identified in the theoretical background, and the employer focused concern about children’s early aspirations, we present a panoramic overview of the changes in individuals’ aspirations over a 3-year period. The paper addresses two research questions: RQ1 How do the career aspirations of individuals change during primary school through a lens of their gendered choices? RQ2 What is the value of the career development theories developed from research with older children and adults in considering the factors affecting the career choices of younger children?

## Theoretical background

Career aspirations can be defined as ‘an individual’s desired goals given ideal circumstances’ (Rojewski, 2007, pp. 88) and can, to a first approximation, be identified in answer to the question: what do you want to do when you grow up?

One early theory about the development of children’s career aspirations was posited by Ginzberg (1952) who split occupational choice into three phases: a fantasy phase before age 11, a tentative choice phase between ages 11 and 17, and a realistic choice phase beyond 17. In the fantasy phase, aspirations are characterised by a child’s belief that they could be whatever they wanted to, making ‘an arbitrary translation of [their] impulses and needs into an occupational choice.’ (Ginzberg, 1952 pp. 492–493). Gottfredson’s (1981) Theory of Circumscription and Compromise provides more detail by which children’s aspirations move from fantasy to realism. In this theory, between the ages of ~3 and 11 years of age children’s early career aspirations are shaped by their developing understanding of size, gender and prestige (circumscription), and from around the age of 14 and up career aspirations are further limited by children’s growing understanding of their own abilities, and by the socially acceptable boundaries set for them by their family and society (compromise). This process produces a unique zone of acceptable career alternatives for each child, within which are jobs they feel comfortable aspiring to (Gottfredson, 2005). In effect, children eliminate careers for their deemed incompatibility between where that career sits within the child’s cognitive ‘occupational map’ and their developing self-image (Gottfredson, 2002).

Perceptions of gender roles are central to both circumscription and compromise, as children learn about and internalise expectations of the types of roles acceptable for men and women (Gottfredson, 1981). Beliefs about gender-specific responsibilities can predict educational and occupational aspirations and choices (Eccles et al., 1983). Understanding of gender roles also influences developing interests and self-efficacy from a young age, for example Bian et al. (2017) found that from age six, girls grouped more boys into the ‘really really smart’ category and began to avoid games introduced as for ‘really really smart’ people. Gender is therefore an important factor within examination of children’s career aspirations.

**Fig. 1:**
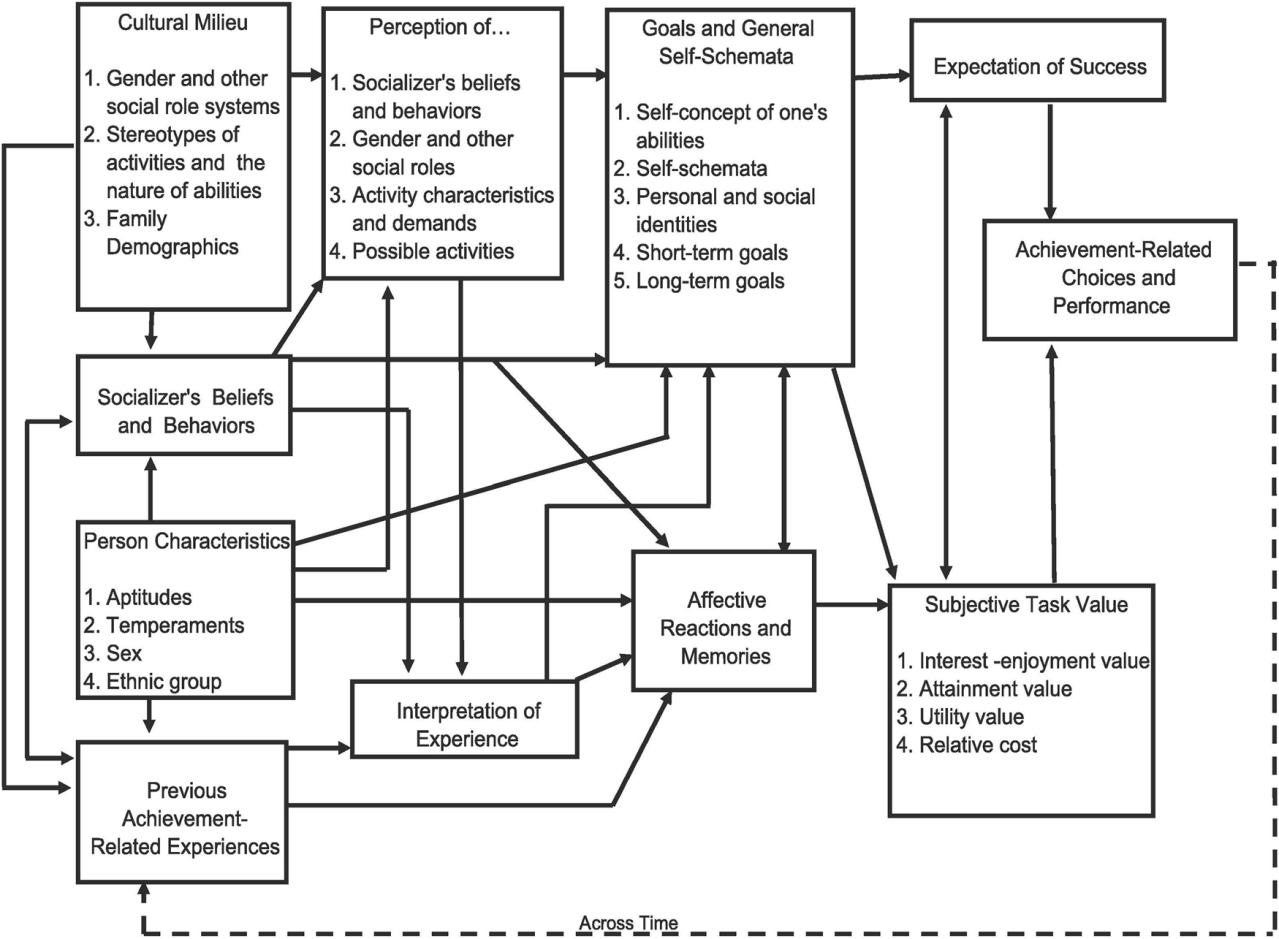
Situated Expectancy Value theory model of achievement choices (from Eccles and Wigfield, 2020).

To consider the broad range of influences on the attainment of a career Eccles and Wigfield (2020) use a situated expectancy-value theory (SEVT) model (Fig. 1). Beginning with an individual’s personal characteristics, the model is a ‘broad theoretical framework that can be used to guide comprehensive programs of research on the both the long-term ontogeny of the beliefs and memories underlying individuals’ motivated achievement-related choices and the more proximal psychological processes that operate over short time frames.’ (Eccles and Wigfield, 2020, pg.1). The model brings together ideas from social cognition, developmental sciences, and sociocultural perspectives into one general theoretical framework (Eccles and Wigfield, 2020). Developed from research with adolescents and adults, the model represents the complex interplay between an individual’s personal characteristics, the beliefs and behaviours of those around them (socialisers e.g. family members, teachers etc), the cultural context in which they find themselves, and the individual’s experiences. Gender has an explicit focus within the SEVT model since initial explorations of it looked at why women made the educational choices they made, as distinct from why they did not make the same educational choices as men (Eccles and Wigfield, 2020).

The complexity of the model represents the complexity of career development and decision making and it should also be considered that ‘all of the processes underlying the SEVT model occur over time and are very much influenced by the immediate situation in which each decision is taking place.’ (Eccles and Wigfield, 2020: pg2). However, the authors do not assume that each category will be activated equally at any given time or across a life-time (Eccles and Wigfield, 2020).

Another factor thought to influence children’s career aspirations is that of role models, which can be defined as ‘individuals who can positively shape a student’s motivation by acting as a successful exemplar’ (Gladstone and Cimpian, 2021, pg. 2). Drawing on expectancy value theory, the motivational theory of role modelling (Morgenroth et al., 2015) identified that the perception of role models could provide goal embodiment, attainability and desirability of particular careers for those developing their career aspirations. These outcomes sit within the ‘cultural milleu’ and ‘perceptions of…’ boxes in Fig. 1.

As well as developing career aspirations with age, children gain a more sophisticated understanding of careers and the process through which an individual might attain different careers over time. Howard and Walsh [2010] outline three broad stages in this development: association (recognise jobs exist, and can make statements about them), sequence (start to link interests with careers and the need to have the ability to do work) and interaction (attaining a career requires the interaction between personal, environment and societal factors). In their study they found that the majority of children aged 5–6 years were in the association stage, children aged 9–10 years were mainly in the sequence stage, and young people aged 13–14 years were in the interaction stage, but didn’t necessarily recognise that systemic societal factors that might influence attainment of careers.

Much research into young people’s career aspirations compares age cohorts of young people. Whilst there are some longitudinal studies that have tracked individuals over time (Baker et al. 2014, Archer et al., 2023), how individual children’s career aspirations change during the course of their early schooling (between ages of 7 and 11) has not received much attention in the literature.

Children’s aspirations for science, technology, engineering and maths (STEM) careers are of particular interest to governments and companies due to shortages within the skilled STEM workforce, and the persisting lack of diversity in those STEM workforces (see e.g. Deloitte, 2018; Engineering UK, 2018). Of particular focus is the continued gendered and classed composition of the physical sciences, technology and engineering fields where white, middle-class males continue to predominate (Cheryan et al., 2017; Engineering UK, 2022). Traditional gender role beliefs are thought to go a long way in explaining the variation between the physical sciences and engineering occupational choices of males and females (Dicke et al., 2019). The limited knowledge that children and young people have of different occupations and sectors has also been suggested as a possible factor in the skills shortage (Weaver, 2023), with further embedding of careers information in the curriculum offered as a possible solution (Reiss and Mujtaba, 2017).

Understanding the natural changes in children’s career aspirations for different sectors as they age, and how they may change as a consequence of an intervention, are important first steps in considering how the lack of diversity in STEM workforces might be addressed. This paper looks at those changing aspirations in STEM, but also in other sectors, to understand the broad sweep of children’s aspirations.

### Methods

This research study is developed from data collected as part of STEM outreach project called Exploring Extreme Environments initiated by Northumbria University. The project was planned as a three-year project of sustained engagement with eight primary schools in the North East of England, with the long-term aim of improving participation in STEM. Potential schools in the region were identified using ‘percentage of children claiming free school meals’ as a proxy for deprivation^1^, and those schools with a percentage higher than the regional average were contacted by email to invite them to take part in the project. Eight schools accepted the invitation.

During the project, schools could choose from eight career aspiration-focused activities including assemblies, in-class workshops, after-school family activities, family story-time, a teacher-led career activity, and teacher professional development (PD) sessions. Most of the activities were delivered by the project staff, although teachers in the schools were supported to include careers information in their science lessons using simple resources through the PD. One of the aims of the project was to broaden children’s awareness of careers over the course of the three year project through these activities which presented a range of STEM careers.

The project started in September 2018 and during the first year of the project all schools completed all the planned activities and scheduled the full offer for the second year of the project in the 2019/20 academic year. However, the second year of the project coincided with the start of the COVID-19 pandemic. In March 2020 all schools in England were closed to most pupils (Department for Education, 2020) and the nature of the project activities, implementation timeline and evaluation were changed to take this into account. The enforced interruption in the planned delivery of the project means that the data presented in this paper are not treated as a measure of impact of the project, but as a source of aspirations data across the period of the Covid pandemic which shed light on children’s career aspirations over time.

### Participants

The eight primary schools in the intervention project were in areas of deprivation and had a percentage of pupils claiming free school meals higher than the regional average of 19.4% in 2018 (Average: 35.8%; Range: 23.1–65.9%). The number of pupils in the schools ranged from 236 to 689 and all schools were co-educational. Four of the schools agreed to be representative ‘evaluation schools’.

The research received ethical approval from Northumbria University. Consent for participation in the research was provided by each school’s headteacher acting in loco parentis, and legal guardians were provided the opportunity to withdraw their children from the research activities (but not the project activities) if they wished. All pupils aged between 7 and 11 present in the evaluation schools on the data collection days in 2019 giving assent to participate, and whose parents had not withdrawn them from the research study, were surveyed. Data from the 2019 sample are reported elsewhere (Padwick et al., 2020b). Only three schools completed a second survey in 2021, and some classes were absent due to covid when the final survey took place. The full dataset of pupils was 621 pupils in 2019 (334 boys, 282 boys, 4 not given) and 300 pupils in 2021 (159 boys, 133 girls, 8 not given).

For the current paper, a sub-set of the full dataset was used. This consisted of 78 pupils (43 girls, 35 boys) who completed the pupil survey in 2019 and 2021, and whose data could be matched across the two time points. The pupils were in Year 3 or 4 (ages 7–9) in 2019 and in Year 5 or 6 (ages 10–11) in 2021. To establish similarity between the full dataset and the matched sample the frequency of jobs coded in each major UK Standard Occupational Classification (SOC) (ONS, 2023) named by these sample in 2021 was compared with the frequency in the larger 2021 dataset. The percentages of jobs in each SOC code differed between data collection points, but the rank order of the most named SOC categories remained constant (SOC3 Associate professionals > SOC2 Professional occupations > SOC6 Caring and leisure occupations). This provided confidence that the children in the sub-sample were similar in their job aspirations to the children in the larger dataset.

### Data collection tools and analysis

The research design is mixed methods and takes a person-centred approach (Berger et al., 2019) to allow for focus on individual differences, rather than treating the cohort just as a single group. Evaluation data from the intervention was collected using a worksheet completed by children. In 2019 the worksheet was administered by a member of the research team who read out each question whilst the children wrote their answers down. Children were informed that there were no right or wrong answers, and that the team were interested in their own thoughts. The data collection lasted between 20 and 30 min. In 2021, due to Covid restrictions, copies of the worksheet were sent to the school and administered by the class teacher. Teachers were given the following instruction ‘Each child should work on their own, and give their own answers. If they need help reading the questions, please assist them, but don’t suggest answers to the questions for them’. The worksheet contained quantitative questions about their enjoyment of, and ability in, science using Likert scales; qualitative free response text asking about three possible job aspirations and reasons for wanting that job; a pupil view template (described elsewhere) and demographic information. The current paper presents findings from the free response text about job aspirations and reasons for wanting those jobs. Children’s responses produced written text in the form of short, individual words, phrases and sentences. A panoramic examination of factors of influence on children’s occupational aspirations was chosen as it was a good fit for this data type (Braun and Clarke, 2022).

Worksheet responses from the larger sample in both 2019 and 2021 were tabulated and variations in spelling of jobs cleansed. Job titles were regularised for analysis e.g. *police*, *police man*, *polis* were all recorded as *police officer*. Where there was ambiguity in the word, the children’s reason for wanting the job was used to provide additional clarity where possible. Some responses were not coded because they did not refer to paid employment or a standard job (such as *mam, dad*, *robber*, *helpful*, *happy man*, *shoes*) and some were indecipherable due to poor handwriting.

Initial job choice analysis was informed by the theories of Ginzberg (1952) and Gottfredson (1981) to look at the range and nature of the aspirations given for the cohort, and on the changes over time for individuals. Job aspirations were coded according to the UK Standard Occupational Classification (SOC) (ONS, 2023) for major, sub-major and sub-minor levels, and a frequency analysis was carried out. Data from children who chose not to give their gender are not included in the separate results for girls or boys, but are included in the broader cohort results.

The first named jobs were also coded into broad categories based on the job title and the sub-minor SOC code. These categories were STEM (e.g. physical sciences, engineering, skilled trades), Health (e.g. doctor, nurse, paramedic), Animal Care (e.g. vet, dog walker), Creative (e.g. youtuber, singer, dance teacher), Sports (e.g. footballer, coach) and Other (e.g. armed forces, shopkeeper, hairdresser). These categories were chosen because they represent areas of concern by employers (STEM, Health), or were highly prevalent in children’s responses. Sankey diagrams are often used in physics and engineering to show flow of energy or materials through a system across different time points. However, they have also been used as a more general data visualisation tool (see e.g. Lamer et al., 2020). A Sankey diagram (Sankeymatic, 2024) was created showing the changes between the first named jobs for children in 2019 and 2021. To establish the spread of aspirations given by each child, the difference between the number of job aspirations given and the number of categories those jobs were in was calculated. We also calculated the changes in STEM, creative or sport careers for individual children using the (up to) three named jobs as a measure of stability or mutability of each child’s career aspiration over time.

To explore the concordance between the reasons pupils’ gave for their aspirations and factors within the SEVT, pupils’ reasons for job choice were analysed using thematic analysis (Braun and Clarke, 2021) following a phronetic iterative approach (Tracy, 2018). Initially the CCCA framework (Howard and Walsh, 2010) was used to develop an inductive codebook. This initial codebook was extended after first analysis to include a broader range of codes related to reasons for choosing careers, and the data were re-coded using this new deductive codebook. Situated expectancy-value theory (SEVT) (Eccles and Wigfield, 2020) was used to categorise the identified codes. Finally, the codes were grouped into themes which captured the underlying similarities between them. These themes were self-actualisation, altruism, characteristics of the job, and role model.

Quantitative analysis was conducted in Excel and IBM SPSS Statistics 28 (IBM, 2021), and qualitative analysis using NVivo 14 (Lumivero, 2023).

## Results

We first present findings on the number and nature of the children’s career aspirations as a cohort in 2019 and 2021 by gender.

### Job aspirations

There were similar numbers of aspirations given by the children in the matched sample in 2019 (all *n* = 166; boys 70; girls 96) and 2021 (all *n* = 160; boys 71; girls 83), and the average number of aspirations given (2) was the same at both time points. In 2019, only 2 children (3%) gave no aspirations, with all other children giving 1 or more aspirations (1:31%; 2:23%; 3:31%). Similarly in 2021, only 2 (different) children gave no aspirations and the remaining children naming at least 1 aspiration (1:29%; 2:28%; 3:40%). The change in the number of aspirations for each individual child between 2019 and 2021 was calculated and showed some variability with 45% of pupils naming the same number of aspirations, 25% naming more, and 31% naming fewer jobs in 2021 than in 2019.

Table 1 shows that the types of jobs named by children in 2019 and 2021 are predominantly in two major SOC codes (3: Associate professionals and technical occupations and 2: Professional occupations) and the overall percentages are similar at each timepoint.

**Table 1:** The percentage of jobs in major SOC categories named by all matched children, and by boys and girls.

SOC category	All children %		Boys %		Girls %	
	2019 (*n* = 165)	2021 (*n* = 159)	2019 (*n* = 69)	2021 (*n* = 73)	2019 (*n* = 96)	2021 (*n* = 86)
1 Managers, directors and senior officials	4.8	4.4	7.2	2.7	3.1	5.8
2 Professional occupations	29.7	22.6	17.4	23.3	38.5	22.1
3 Associate professionals and technical occupations	50.3	57.2	62.3	61.6	41.7	53.5
4 Administrative and secretarial occupations	0.6	0.6	0.0	0.0	1.0	1.2
5 Skilled trade occupations	5.5	6.9	8.7	8.2	3.1	5.8
6 Caring, leisure and other service occupations	7.3	6.3	2.9	1.4	10.4	10.5
7 Sales and customer service occupations	0.6	1.9	0.0	2.7	1.0	1.2
8 Process, plant and machine occupations	1.2	0.0	1.4	0.0	1.0	0.0

The number of distinct jobs named by children was greater in 2021 (*n* = 92) than in 2019 (*n* = 71). At both timepoints the two most frequently named job for boys were footballer (37% in 2019 and 31% in 2021) and police officer (23% in 2019 and 14% in 2021). There was more variability and broader range of jobs named by girls when comparing between 2019 and 2021 (Table 2).

**Table 2:** The most commonly named jobs from the matched sample of pupils (*n* = 78) in 2019 and 2021.

Rank	Boys		Girls	
	2019	2021	2019	2021
1	footballer (37%)	footballer (31%)	teacher (28%)	actor/actress (23%)
2	police officer (23%)	police officer (14%)	vet (12%)	vet (12%) nail technician (12%)
3	builder (14%)	scientist (11%)	youtuber (16%)	artist (9%) teacher (9%) author (9%)
4	army (11%) firefighter (11%) shopkeeper (11%)	youtuber (9%)	doctor (12%) hairdresser (12%)	baker (5%) chef (5%) detective (5%) doctor (5%) hairdresser (5%) lawyer (5%) nurse (5%)

**Fig. 2:**
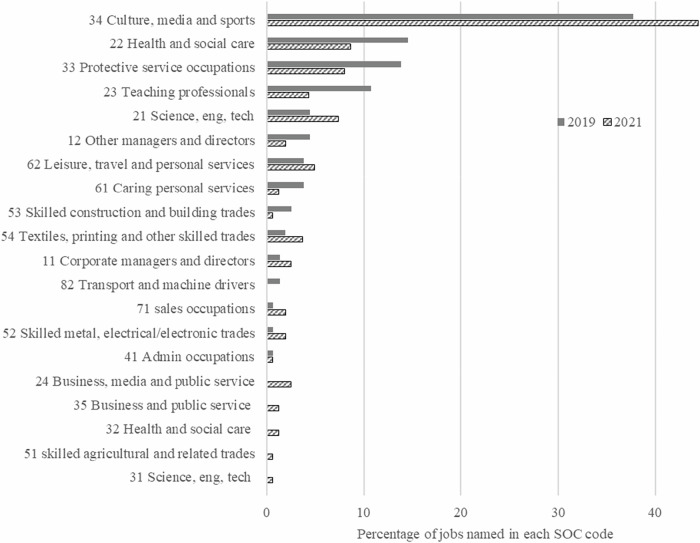
Comparison of the percentage of jobs in SOC sub-major groupings in 2019 and 2021.

Looking at the cohort as a whole, a broader range of SOC categories were given in 2021 (Fig. 2) with aspirations from five new SOC codes. Some children also gave more detailed or specific job titles in 2021 e.g. child who wrote ‘doctor or nurse’ in 2019 wrote ‘radiographer’ in 2021, and child who wanted to ‘drive an ambulance’ in 2019 wrote ‘paramedic’ in 2021. SOC 34 (Culture, media and sports occupations) was the most popular classification including footballer (21%), youtuber (14%), other professional sports person (13%), and performer (13%) being given in 2019; and footballer (18%), actor (14%), artist (14%), other professionals sports person (14%) in 2021.

There was a level of stability of aspirations over time for some pupils with 38% of pupils having at least one aspiration the same in 2019 and 2021. However, 60% of pupils also changed at least one of their career aspirations between the two data collection points.

The sub-major SOC codes indicate the spread of aspirations given by an individual child i.e. were all of their aspirations in a similar area, or were their aspirations in different sectors? At both time points a similar number of children gave more than one aspiration (52 in 2019 and 53 in 2021). Of those children, only 10 children in 2019 and 8 children in 2021 had all their aspirations in only one sub major SOC code (13% and 10% of the sample respectively). The remaining children had aspirations in two (2019: 33%; 2021: 45%) or three (2019: 21%; 2021: 13%) sub-major SOC code categories.

Comparing the change in the breadth of aspirations for the 25 children who had the same number of aspirations (i.e. 2 or 3 aspirations) in 2019 and 2021, we find that in 2021 there were 9 (36%) children who had aspirations in more categories, 10 (40%) children had aspirations in the same number of categories, and 6 (24%) had aspirations in fewer categories.

**Fig. 3:**
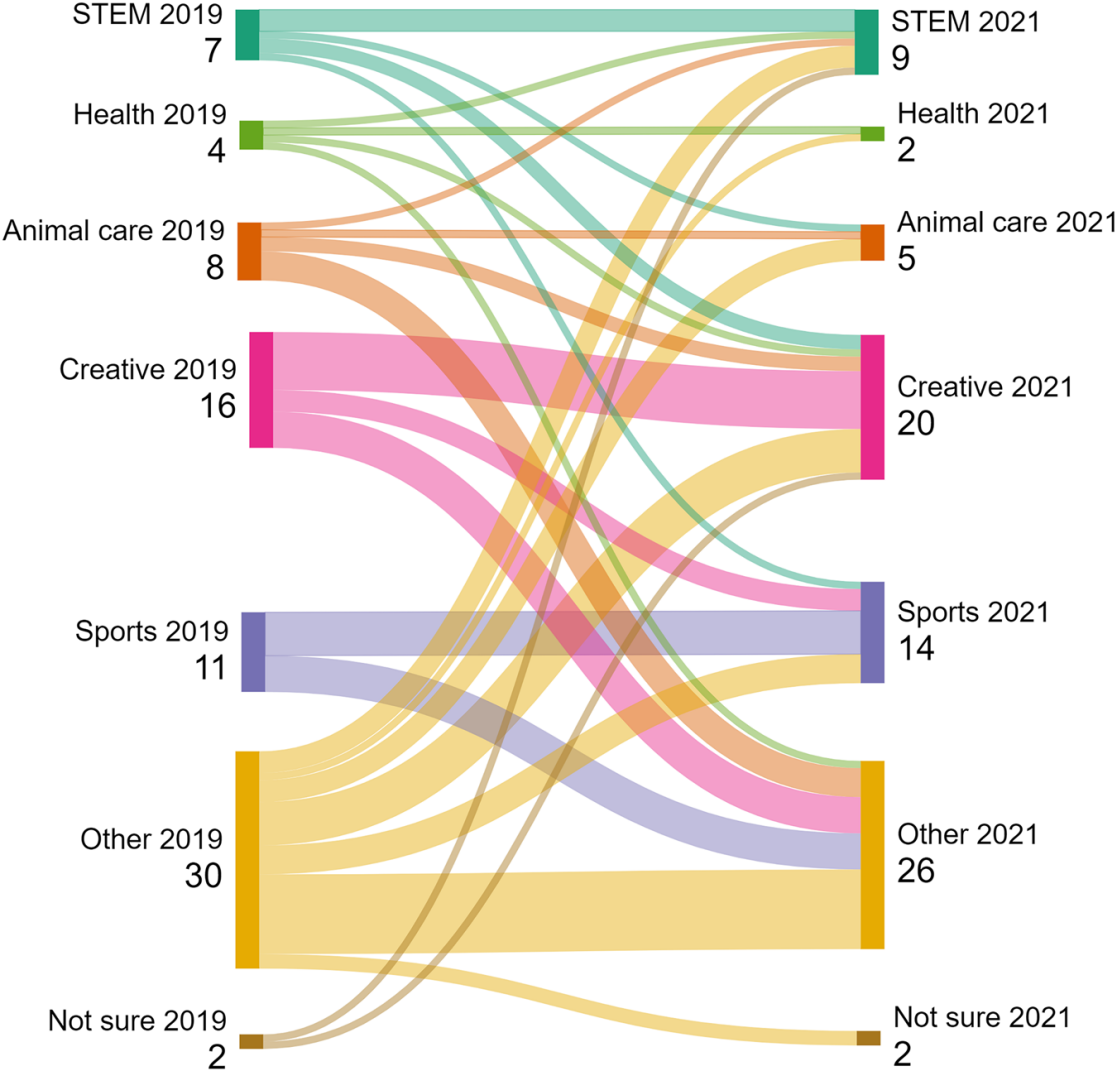
Sankey diagram showing the changes in children’s broad aspirations between 2019 and 2021 using children’s first named jobs.

Figure 3 provides an indication of the amount of flux and stability for the first named job at each timepoint using broad categories of jobs, showing a proportion of each category remaining the same or changing to other categories.

### Reasons for job choices

We next present findings related to children’s job choices and how these relate to factors in the SEVT (Eccles and Wigfield, 2020) for the cohort. Thematic analysis identified four themes that brought together the different codes (Table 3).

**Table 3:** Themes identified in the data, the related codes assigned to children’s reasons for wanting to do a particular job and the links to factors included in the SEVT (Eccles and Wigfield, 2020).

Theme	Deductive code	SEVT category
Self-actualisation	enjoyment	interest-enjoyment subjective task value; affective reactions and memories
	have skill already	expectancies for success; affective academic self-concepts; self-concepts of one’s abilities
	personal development	attainment subjective task value
Altruism	to help people	affective reactions and memories
	to help animals	
	to help country	
Characteristics of the job	money	utility subjective task value
	magical connection	perception of possible activities
	quality of person doing the job	perception of socialisers’ beliefs and behaviours
	looks interesting	interest-enjoyment subjective task value
	pure association	perception of activity characteristics and demands
	job statement	perception of activity characteristics
Role model	role model	perception of socialiser’s beliefs and behaviours; cultural milieu previous achievement-related experiences

The first theme is ‘self-actualisation’ wherein reasons given relate to personal flourishing, either now or in the future. Many responses coded/categorised in this theme spoke about children’s current enjoyment and current skills in activities linked to the career. Jobs, particularly in the creative, media and sports sectors, were often linked to hobbies that the children already had, and the pleasure that they found in those activities.

*because I love to dance and do it every day and I go to dance lessons* (dance teacher)

*I do boxing and enjoy it* (boxer)

*make food at home and feed people* (baker)

*because trains are my favourite thing* (train driver)

There was also an awareness, in some responses, of the emotional benefits of the activities they already did, and by extension, the benefit that would accrue from the job.

*because when I am acting I feel happy* (actor)

*because I love doing it and it is really calming* (artist)

Future personal development was also included in the self-actualisation theme with children giving reasons which related to improvement and challenge.

*because I like to challenge myself* (youtuber)

*because I want to be smart* (scientist)

*because I want to be good at it* (footballer)

*because I really like to learn different things* (teacher)

*I like anime and want to learn Japanese* (Manga editor)

The second theme identified was ‘altruism’. Reasons in this theme related to helping, whether that be other people, animals or society as a whole.

*to have money for the homeless* (shopkeeper)

*to help people with knowledge* (teacher)

*because I love animals and want to save them* (vet)

*to keep the world safe* (policeman)

Within the third theme, ‘characteristics of the job’ reasons for job choice were related to the observable characteristics of jobs or the people doing them, children’s perceptions of what the job might entail, and the financial benefits of the job. Some of the reasons were quite specific and indicated some knowledge of the job.

*put fires out; they save houses* (fire fighter)

*you can do something amazing and save lives, like making a cure for cancer* (scientist)

*get money and give it to my parents* (footballer)

*Sometimes good wages and good shifts* (brick layer)

‘Role models’ was the final theme identified, with children mentioning specific individuals as a reason for wanting a particular job.

*Mam does it (*financer *sic*)

*because my dad bakes cakes and I really enjoy helping him* (baker)

*I love watching my Mum* (nail technician)

*because my grandad was like one* (taxi girl)

Perhaps surprisingly, only two children alluded to non-family members as possible role-models.

*because I watch people and it looks fun* (youtuber)

*because I want to play for Manchester city with Sergio Aguero*. [footballer]

Interestingly, different children gave a range of different reasons for the same job showing the different motivations present. For example, for police officer children gave reasons which included: role models in their family ‘*Grandad was one*’; altruism ‘*to help people*’, ‘*to protect people*’ ‘*to save people’s lives’*; skills and attributes that they have ‘*because I am fast strong always ready*’, and the activities that the job might entail ‘*because I get to go fast’, ‘to arrest bad people’*. Comparing the reasons with the factors in the SEVT it can be seen that they relate to different factors as highlighted in Table 3, for example ‘role model’ relates to ‘perception of socialiser’s beliefs and behaviours’, ‘cultural milieu’, and ‘previous achievement-related experiences’, and ‘altruism’ relates to ‘affective reactions and memories’.

Looking at the reasons given in 2019 there were no time-related descriptions included, but in the 2021 reasons there are some allusions to the time spent doing the activity e.g. ‘*I’ve played it all my life*’, ‘*I have ridden from a young age, I was good at it and my horse was confiden*t’, ‘*Been going to acting school for a year and really like it*’. This suggests that some children may be progressing to a ‘tentative realist’ view of possible jobs (Ginzberg, 1952).

### Changing aspirations for individual children

We next present findings for individuals which explore how their aspirations changed over time. These are presented for three different sectors: STEM, cultural and sport.

#### STEM

Figure 2 shows that there were changes to the broad category of first-named STEM aspirations written by children between 2019 and 2021. To explore RQ2 the children who had STEM aspirations in 2019 were considered in more detail across all of the aspirations they gave, not just the first one named.

In 2019, 31 pupils (40%) gave a STEM aspiration with more girls than boys naming a STEM career. However, over time there was a differential change in the STEM aspirations of girls and boys, with the percentage of girls decreasing and the percentage of boys increasing (Table 4). Looking in detail at the 22 girls who gave a STEM aspiration in 2019, 11 of these wanted to be a vet, 9 wanted to work in medical STEM (doctor/nurse/dentist), and 2 named other STEM careers (astronaut/scientist). By 2021, there were only 8 girls who still named a STEM job and they were mainly in the same areas (4 vet, 3 medical STEM, 1 geologist).

**Table 4:** Changes in children’s STEM aspirations between 2019 and 2021.

Measure	All (*n* = 78)	Boys (*n* = 35)	Girls (*n* = 43)
STEM aspiration in 2019	31 (40%)	9 (26%)	22 (51%)
STEM aspiration in 2021	28 (36%)	14 (40%)	14 (33%)
STEM aspiration in 2019 AND 2021	14 (18%)	6 (17%)	8 (19%)
Gained STEM aspiration by 2021	14 (18%)	8 (23%)	6 (14%)
Lost STEM aspiration by 2021	17 (22%)	3 (9%)	14 (33%)

Of the 14 children (18% of the sample) who gave a STEM aspiration in 2019 and 2021, 10 children had the same or similar aspirations at both times (e.g. fixing boats became mechanic), and 4 children changed aspirations within the STEM classification (e.g. vet became geologist). In 2019, the majority of girls’ STEM aspirations were in medical and biological sciences and the majority of boys’ STEM aspirations were in physical sciences, engineering and technical sectors. In 2021, there was a broader range of specific STEM aspirations, but still broadly medical and biological sciences for girls, and physical sciences and engineering fields for boys.

#### Creative

The creative careers category (sub-major SOC code 34) includes the fields of music and performance (e.g. singer, drummer, dancer), acting, writing (e.g. author, manga editor) and visual arts (e.g. artist, movie creator, illustrator). In 2019, 24 children named at least one creative career (6 boys, 18 girls) and by 2021 this had increased to 33 children (10 boys, 23 girls).

The named careers also included youtuber/streamer. In the matched sample 10 children (13%) in 2019 and 4 children (5%) in 2021 named youtuber (or similar) as a possible career. The children were different at each time point i.e. all those children who named youtuber as a possible career in 2019 did not give that as a career in 2021, and vice versa.

#### Sports

Children named a range of different sports-based careers including swimmer, wrestler, dressage rider, cricketer, gymnast, and sports coach. In both 2019 and 2021 there were 23 children who gave at least one sports aspiration although they were not the same children in each case. Between 2019 and 2021, 13 children retained a sports aspiration, but 10 children no longer gave an aspiration for sports (4 boys, 6 girls) and 10 gained an aspiration for sports (4 boys, 6 girls).

The most popular sports job at both timepoints named by boys was footballer (soccer player), with the aspiration to footballer being relatively stable for boys, but not for girls (Table 5).

**Table 5:** Changes in children’s football aspirations between 2019 and 2021.

Measure	All (*n* = 78)	Boys (*n* = 35)	Girls (*n* = 43)
Football aspiration in 2019	16 (21%)	13 (37%)	3 (7%)
Football aspiration in 2021	15 (19%)	12 (34%)	3 (7%)
Football aspiration in 2019 AND 2021	11 (14%)	11 (31%)	0
Gained Football aspiration by 2021	4 (5%)	1 (3%)	3 (7%)
Lost Football aspiration by 2021	5 (6%)	2 (6%)	3 (7%)

## Discussion

CIAG in schools in England has generally focused on pupils aged 13–18. Yet career development theories, which map the development of career aspirations from pre-school to adulthood and beyond, show that career aspirations are developed earlier, meaning that pupils will have formed career aspirations already in primary school (before age 11). Knowledge of the stages of career development is essential for tailoring CIAG to meet the needs of all pupils throughout their schooling.

### Development of aspiration

In the first research question we asked how career aspirations of individuals change during primary school through a lens of gendered choices. In exploration of this we worked with existing career development theories. These outline children’s journeys in careers understanding and aspiration, beginning from early knowledge about jobs and who does them (Howard and Walsh, 2010), the early development of fantasy aspirations (Ginzberg, 1952; Gottfredson, 1981) and the progressive tailoring of aspirations based on their growing awareness of the world and how they fit in it (Howard and Walsh, 2010; Gottfredson, 1981). This study has looked at the developing aspirations of children aged 7- to 11 in primary education.

Nearly all children within this study held aspirations for future occupations. At both timepoints, 97% of the children in the study were able to name at least one future aspiration. At ages 7–9 children’s aspirations were closely tied to what they have seen and what they know. The large majority of aspirations were grouped within SOC code 34 the category for sports, culture and media jobs, which encompasses popular aspirations for footballer, youtuber and social media and actors/actresses. SOC 34 aspirations accounted for 38% of aspirations in 2019 and 44% of aspirations in 2021. The findings support the framework posited by Ginzberg (1952), with many children in this study showing that their early career aspirations are shaped by play in the fantasy phase. Aspirations have parallels with common interests, hobbies and pastimes of children of this age. This is also supportive of children entering Howard and Walsh sequence stage where they start to link their interests with possible careers (Howard and Walsh, 2010).

### Flux in aspirations

Few studies about children’s aspirations have been longitudinal. An important feature of this study was the opportunity to compare stability of aspirations across the primary school age for the same individuals in a group of children. We asked children to provide aspirations in 2019 when they were 7–9 and in 2021 when they were 10–11. Our analysis identified some stability in children’s aspirations. For some children (38%) there is stability in their wider career aspirations, and they either have at least one aspiration the same, or have a closely related or advanced version of the same aspiration, as they did two years prior. However, the study finds that between ages of 7/9 and 10/11 there is significant flux in children’s career aspirations with over 60% of children changing at least one of their (up to three) career aspirations over the space of 2 years. This flux is also seen in the first named jobs given by children (Fig. 3) as children change between broad sectors e.g. from STEM to animal care, creative, sports and other categories. It is noticeable in Fig. 3 that although there is change between broad groups, in terms of first named jobs, no child who named a creative or sports job in 2019 changed that aspiration to one which could be broadly categorised as STEM (including health and animal care) in 2021. Conversely, children who named a job in the broader STEM categories, did change their first-named aspiration to ones in creative, sports or other categories.

The stability of aspiration (38%) is lower than found by Atherton et al. (2009), where 65% of children aged 11–12 self-reported that they had had the same career aspiration for over two years. That level of certainty and stability is not seen for the majority of children in the current longitudinal study and could be a consequence of not relying on self-reporting of children’s recollection of how long they had held a particular career aspiration.

Contrary to two earlier surveys of young people (British Science Association, 2020; ASPIRES Research, 2020) which found an increase of interest in healthcare careers during the pandemic, in the current sample there is no indication that younger children’s experience of the pandemic increased their interest in healthcare careers, although there was a small increase in the number of children interested in science.

### Gender differences in aspiration

The career development theories drawn on within this study acknowledge the prominent influence of gender roles on children’s occupational choices (Gottfredson, 1981). Social and cultural values about gender define the types of roles, responsibilities and behaviours deemed acceptable for boys and girls (Dicke et al., 2019). While the girls and boys in this study aspire to jobs at similar levels and within similar SOC sectors, our study found notable gender differences in type of aspiration. There is a clear disparity in the jobs aspired to by boys and girls with footballer being the most named job for boys in both 2019 and 2021, and teacher (2019) and actor/actress (2021) being the most named jobs for girls. It is particularly apparent when looking at STEM jobs, where girls mainly aspired to veterinary or medical STEM, and boys aspired to physical sciences and engineering jobs. This corresponds with other studies that have found that physical science and engineering jobs code masculine and biology and healthcare careers code feminine (Archer et al., 2013). We also see changes in the proportion of girls and boys aspiring to STEM careers over time. In 2019 over half of the girls gave a STEM aspiration, but this decreased to 33% in 2021. For boys, in 2019 there were 26% who gave at least one STEM aspiration, which increased to 40%. This is clear evidence that children are both learning about, and internalising, the type of roles and responsibilities that are socially acceptable for their gender (Goffredson, 1981).

### Methodological implications

Many studies of children’s aspirations ask children only for a single possibility (see e.g. Atherton et al., 2009; Chambers et al., 2018; Sheldrake, 2020). However, the method used in the present study was to ask children to name up to three aspiration choices. The 78 children named a breadth of career aspirations within their choices, often from different sectors within the SOC codes which shows that, at this age, many children still have not narrowed their interests and aspire to careers in different sectors. Even where named jobs all fell within the same sub-major SOC code, looking more closely showed that, even in some of those cases, the jobs were different e.g. author and Olympic swimmer (sub-major SOC code 34); police officer and fire fighter (sub-major SOC code 33) and games designer and scientist (sub-major SOC code 21).

Our study has shown the majority of children could name more than one possible job that they could see themselves doing. Consequently, studies asking children to name only one possible career aspiration could lead to under-reporting of the range of children’s career aspirations, and the authors recommend that other studies follow a similar practice of allowing children to give more than one possible job. The data indicate that children will still name only one aspiration if that is the only job they want, but it provides opportunity for children to express their broader understanding of their possible selves (Markus and Nurius, 1986).

### Valuable additions to career development theories

Our second research question explored the value of career development theories developed from research with adolescents and adults in identifying factors affecting career choices of younger children. This study incorporates the motivational theory of role modelling into career development theories and the study of aspirations. According to model presented by Morgenroth et al. (2015) role models are assumed to provide goal embodiment, attainability and desirability of careers. Previous research into role models has tended to focus on adolescents and young adults (Gladstone and Cimpian, 2021), and we extend this to a younger age group. The current research indicates that for younger children the most significant role models are family members, with children referring to watching what a family member does or writing about the precedents for a particular career within their family. With only a few exceptions, the children in the current research did not identify non-family members as their role models. Of course, as adults, when looking back at the career path they have chosen they may identify people other than family members as role models, but at this point in their lives, the concept is not strongly present in their reasoning for a career. This highlights the significant influence of cultural milieu, particularly family demographics and family beliefs and behaviours in the development of aspirations, rather than outside influences. It also suggests that the use of role models to encourage children towards a particular career is more complicated than perhaps indicated in current discourse, and that further research into younger children’s understanding of role models as significant adults is needed.

As well as looking at children’s aspirations and how they develop, this study incorporates motivation for different careers. Working with the SEVTs achievement related motivations we mapped children’s motivations to do their chosen aspiration to categories within the SEVT. Although developed with older adolescents and adults, factors within the SEVT (Eccles and Wigfield, 2020) can be seen in the reasons given for children’s aspirations (Table 3) albeit in a nascent fashion. In analysis of children’s reasons for wanting to do a particular job we identified self-actualisation, altruism, characteristics of the job and role models, all which could be mapped to categories within the SEVT. In the aspirations of younger children for police, army, firefighter, teacher, vet and doctor we find altruism is strong motivation, where young people’s achievement-related motivations are focused on their affective reactions and memories and doing good (Eccles and Wigfield, 2020). We found that two years later some altruistic focused roles remain popular among boys (police officer) and girls (vet, teacher) however the dominance of this motivation is reduced, new jobs emerge, and the variance of jobs increases.

The value of incorporating the SEVT alongside career development theories is that it allows us to see beyond demographic differences, such as gender categories, and to begin to interrogate the wider range of influences on children’s motivations for occupations. Even among children with the same aspiration, we identify different motivations and drivers of these ambitions in individuals, including affective academic self-concept, self-efficacy and affective reactions and memories. Embedding understanding of achievement related motivations can therefore support us to better understand the variation in both children’s aspirations and motivations. Looking at the reasons behind aspirations also provides us with greater evidence of career development stages. For example, reasons they give for choosing the job indicate that they have not considered how they would attain that job but currently enjoy the activity e.g. Lifeguard*: I feel very enthusiastic about it. I like swimming at [the] baths*. Similarly, there are indications that some of the children in the sample in 2021 are starting to recognise that effort is important in the attainment of aspirations, as indicated by their reasons including a time-related element. This suggests that they are moving from a fantasy to a tentative realist view (Ginzberg, 1952).

While the SEVT was designed with adolescents in mind, we find evidence of the applicability of the categories and constructs within it among younger children. Eccles and Wigfield did not assume that all constructs in the SEVT would be applicable at any given time or life stage. Career aspirations and career reasons, or motivations, can support each other in presenting a broader picture of how children work through and progress understanding of their future selves. Further research could consider how active each category of the SEVT along an age-linked timeline in a similar way to career development theories, and could explore the development of research tools to explore the motivations behind young children’s career aspirations in more detail. Equally, the SEVT has utility in considering how children could be supported to achieve their aspirations, in a similar way that it is used for young adults (see e.g. Xu and Lastrapes, 2022).

### Implications for policy and practice

This study has shown how children’s aspirations are shaped by the job roles that they see around them, and that they have some knowledge of. Many of the aspirations named in the current study are linked to children’s daily life such as teacher, healthcare worker, police officer, sports player and coach, and entertainment professional. Very few of the roles named are what might be considered to be ‘office based’. The narrow range of aspirations seen in the current study, and in previous studies on older children, has led some organisations to express concern about the disconnect between young people’s aspirations and the job market (Rogers et al. 2020). However, the theoretical frameworks about the development of children’s career aspirations predict that this disconnect is part of the process of career exploration. As children develop, both Ginzberg (1952) and Gottfredson (2002) indicate that a level of realism sets in as children come to understand that not all jobs may be obtainable or desirable, and Howard and Walsh (2010) outline different steps within the career development process. That the most popular jobs were within SOC category 34 ‘culture, media and sports occupations’ and directly link to children’s hobbies is not a surprise. CIAG that is tailored for the fantasy (Ginzberg, 1952) or sequence development stages (Howard and Walsh, 2010) present in young children in primary/elementary school will recognise the dominance of these type of aspirations, and support children to progress or expand their thinking. For example, the likelihood of a child achieving a job in football is small and so there is opportunity for sports organisations to support children’s developing career aspirations beyond this narrow focus by showcasing the breadth of sport-adjacent careers which are required for a single professional level footballer (or other sports) to succeed e.g. groundskeepers, coaches, physiotherapists, dieticians etc. In this way, children’s current interests and aspiration can be used to carefully explore other careers and support them to develop a more realist view of their future jobs.

The reduction in the percentage of girls aspiring to any type of STEM career as they age is still concerning, particularly given the focus in many schools on reducing stereotypes. It emphasises the importance of children’s key influencers (family and school staff) offering a non-stereotyped view of careers from an early age.

Notwithstanding the low likelihood of achieving some hobby-linked career aspirations, when talking with children about their career aspirations, it is important for adults to remember that, for the child, the career aspiration is real, even if it is not realistic. Therefore, any such conversations should be sensitive, exploratory and supportive, rather than e.g. simply pointing out the unlikeliness of achieving the career.

### Conclusion

This is a small study, but one of the first to specifically follow young children’s career aspirations longitudinally as they age. The findings indicate that there is some stability of aspirations over time, but that many children do change their ideas during primary/elementary education. The changeability of aspirations at this young age is, perhaps, to be expected, but provides a strong argument for the introduction of career-related learning in primary school to support young children in their career exploration. It is important to introduce children to a wide range of possible jobs beyond those that they see in their community to support them to keep their zone of acceptable alternatives (Gottfredson, 1981) as broad as possible for as long as possible.

## Data Availability

Data supporting this study are not publicly available due to the nature of the consent provided by participants. Access is subject to approval and a data sharing agreement. Further information on the data and access conditions is available at 10.25398/rd.northumbria.29561747.

## Footnotes

1 In England families can claim ‘Free school meals’ if their income is below a threshold or they are in receipt of certain welfare benefits. Percentage free school meals for a school is therefore commonly used as a proxy for levels of deprivation in the school.
